# Roles of Autophagy in MPP^+^-Induced Neurotoxicity *In Vivo*: The Involvement of Mitochondria and α-Synuclein Aggregation

**DOI:** 10.1371/journal.pone.0091074

**Published:** 2014-03-19

**Authors:** Kai-Chih Hung, Hui-Ju Huang, Ming-Wei Lin, Yen-Ping Lei, Anya Maan-yuh Lin

**Affiliations:** 1 Department of Physiology, National Yang-Ming University, Taipei, Taiwan; 2 Department of Medical Research and Education, Taipei Veterans General Hospital, Taipei, Taiwan; 3 Institute of Public Health, National Yang-Ming University, Taipei, Taiwan; 4 Department of Nursing, National Yang-Ming University, Taipei, Taiwan; 5 Department of Pharmacology, National Yang-Ming University, Taipei, Taiwan; Foundation for Biomedical Research Academy of Athens, Greece

## Abstract

Macroautophagy (also known as autophagy) is an intracellular self-eating mechanism and has been proposed as both neuroprotective and neurodestructive in the central nervous system (CNS) neurodegenerative diseases. In the present study, the role of autophagy involving mitochondria and α-synuclein was investigated in MPP^+^ (1-methyl-4-phenylpyridinium)-induced oxidative injury in chloral hydrate-anesthetized rats *in vivo*. The oxidative mechanism underlying MPP^+^-induced neurotoxicity was identified by elevated lipid peroxidation and heme oxygenase-1 levels, a redox-regulated protein in MPP^+^-infused substantia nigra (SN). At the same time, MPP^+^ significantly increased LC3-II levels, a hallmark protein of autophagy. To block MPP^+^-induced autophagy in rat brain, Atg7siRNA was intranigrally infused 4 d prior to MPP^+^ infusion. Western blot assay showed that *in vivo* Atg7siRNA transfection not only reduced Atg7 levels in the MPP^+^-infused SN but attenuated MPP^+^-induced elevation in LC3-II levels, activation of caspase 9 and reduction in tyrosine hydroxylase levels, indicating that autophagy is pro-death. The immunostaining study demonstrated co-localization of LC3 and succinate dehydrogenase (a mitochondrial complex II) as well as LC3 and α-synuclein, suggesting that autophagy may engulf mitochondria and α-synuclein. Indeed, *in vivo* Atg7siRNA transfection mitigated MPP^+^-induced reduction in cytochrome c oxidase. In addition, MPP^+^-induced autophagy differentially altered the α-synuclein aggregates in the infused SN. In conclusion, autophagy plays a prodeath role in the MPP^+^-induced oxidative injury by sequestering mitochondria in the rat brain. Moreover, our data suggest that the benefits of autophagy depend on the levels of α-synuclein aggregates in the nigrostriatal dopaminergic system of the rat brain.

## Introduction

Macroautophagy (also known as autophagy) is a self-eating mechanism by de novo synthesizing double-membrane autophagosomes which engulf misfolded proteins and cytosolic organelles, such as mitochondria. During the progression of autophagy, LC3-I is conjugated to phosphatidylethanolamine to form LC3-II (a biomarker of autophagy) in an Atg7-dependent manner. LC3-II locates in the inner and outer membranes of autophagosomes. Autolysosomes are formed by fusing autophagosomes with lysosomes to degrade the contents [1]–[3]. A physiological role of autophagy has been proposed for the cellular homeostasis [3]–[4]. However, clinical morphological studies demonstrated marked autophagic features in the brain tissues of patients with CNS neurodegenerative diseases [5]–[6]. Two sides of autophagy have been proposed in the CNS neurodegeneration: neuroprotective and neurodestructive [3]–[4], [7]. Autophagy is considered as a neuroprotective strategy because autophagy is reportedly induced to cope with unfavorable situations, such as serum deprivation and hypoxia [8]–[9]. Furthermore, autophagy induction has been suggested as a therapeutic strategy against CNS neurodegenerative diseases [10]–[11]. Nevertheless, several studies have employed silencing autophagy-related genes to attenuate neurotoxicity [12]–[13], indicating that autophagy is pro-death, referred as a type II programmed cell death [14] in CNS neurodegeneration.

Due to the elevated oxidative stress in the brains of patients with Parkinson's disease [15], a significant body of studies has reported related deleterious events in the etiology of CNS neurodegeneration, including protein aggregation, mitochondria damage, necrosis and apoptosis [13], [16]–[20]. Recently, oxidative stress has been proposed as one of the inducers of autophagy [16], [21]. To support this notion, neurotoxins with oxidative properties were employed to induce neurotoxicity and autophagy as well [6], [9], [11]–[13], [20]. Furthermore, antioxidative treatment is capable of reducing autophagy and cell loss [12], [22], suggesting that oxidative stress is responsible for excessive autophagy in CNS neurodegenerative diseases. Mitochondrion is reportedly one possible target of autophagy [1], [3]. Ultrastructural studies have demonstrated double-membrane vacuoles embracing mitochondria in 1-methyl-4-phenylpyridinium (MPP^+^)-treated neurons [13]. Moreover, autophagy inhibition prevented neurotoxins-induced mitochondria loss [9], [12]–[13], indicating a neurodestructive role of autophagy via degrading mitochondria. However, some studies have suggested that autophagy is neuroprotective by sequestering damaged mitochondria in the CNS neurodegenerative diseases [23]–[24].

α-Synuclein containing 140 amino acids primarily locates in the presynaptic terminal. Physiologically, α-synuclein is proposed to regulate the metabolism of neurotransmitters, including synthesis, storage, release and uptake [25]–[26]. Clinical studies have demonstrated pathological deposition of α-synuclein in the Lewy bodies of substantia nigra (SN) in Parkinsonian patients [27]–[28]. The neurotoxic mechanisms underlying α-synuclein have been investigated, including interfering with normal cellular trafficking [29] and damaging mitochondria [30]. Preclinical studies using Parkinsonian animal models showed MPP^+^-induced α-synuclein up-regulation and/or aggregation, in addition to neurotoxicity [11], [16], [31]–[32]. Several studies have suggested that autophagy induction enhanced α-synuclein degradation [11], [33]–[34]; therefore, up-regulation and/or aggregation of α-synuclein may be due to abnormal autophagy. However, limited *in vivo* studies have focused on the dynamic changes of autophagy involving mitochondria and α-synuclein aggregation in the CNS neurodegeneration [12]. In the present study, a Parkinsonian animal model was used by intranigral infusion of MPP^+^, an active metabolite of 1-methyl-4-phenyl-1,2,3,6-tetrahydropyridine by monoamine oxidase B. MPP^+^ is taken by dopamine transporters and inhibits mitochondrial complex I, leading to neuronal damages [19]. The time-dependent changes in autophagy were delineated in MPP^+^-infused SN of anesthetized rats. The mechanisms underlying MPP^+^-elevated autophagy were investigated by *in vivo* Atg7siRNA transfection to silence Atg7 gene which encodes an autophagy essential protein Atg7, a ubiquitin-activating enzyme protein [1]–[3].

## Materials and Methods

Adult, male Sprague-Dawley rats, weighing 250–350 g, were supplied by the National Laboratory Animal Breeding and Research Center, Taipei, Taiwan, R.O.C.. All animals (3 rats/cage) were housed in an air-conditioned room (22±2°C) on a 12-h light/dark cycle (06:00–18:00 h light) and had free access to food and water. These animals were maintained according to the guidelines established in “Guide for the care and use of laboratory animals” prepared by the Committee on Care and Use of Laboratory Animals of the Institute of Laboratory Animal Resources Commission on Life Sciences, National Research Council, U.S.A. (1985). The use of animals has been approved by the Institutional Animal Care and Use Committee of Taipei Veterans General Hospital, Taipei, Taiwan, R.O.C..

### Surgery and intranigral infusion of siRNA and MPP^+^

Rats were anesthetized with chloral hydrate (450 mg/kg, i.p., Sigma, St. Louis MO) and placed in a stereotaxic instrument (David Kopf Instruments, Palo Alto, CA). After skin incision and exposure of the parietal bone, holes above the cortical surface were drilled for intranigral infusion. Ten picomoles of Atg7 siRNA or control siRNA (Accell SMARTpool, Thermo, Waltham, MA) in 0.5 µl diethyl pyrocarbonate H_2_O were stereotaxically infused through a 30 gauge stainless steel needle into the SN with coordinates of 3.2 mm anterior, 2.3 mm above the interaural zero, 2.1 mm lateral to the midline and 3.5 mm below the incisor bar [35]. The injection needle was held in place for an additional 3 minutes (min) following drug infusion. Four days (d) after siRNA infusion, rats were re-anesthetized for intranigral infusion of MPP^+^ (3 µg/µl, Sigma) at a rate of 0.2 µl/min. After the surgery, rats recovered from anesthesia and were placed in home cages for the indicated times.

### Fluorescence assay of lipid peroxidation in SN

At the end of *in vivo* experiments, rats were sacrificed by decapitation. Substantia nigra was dissected and homogenized in chilled 400 µl chloroform and 200 µl methanol. After centrifugation, an aliquot of the chloroform and methanol layer was measured using a spectrofluorometer (Aminco Bowman-2, U.S.A.). Lipid peroxidation was determined by the levels of malondialdehyde and its dihydropyridine polymers which emit fluorescence at 426 nm when activated by UV at 356 nm [36].

### High pressure liquid chromatography coupled with electrochemical detection (HPLC-ECD) analysis of striatal dopamine content

After sacrifice, striata from both hemispheres were dissected, immediately frozen in liquid nitrogen and stored at −80°C until analysis. To measure striatal dopamine content, an HPLC (CC5/PM80, Bioanalytical Systems Inc., West Lafayette, IN) coupled with electrochemical detection (LC-4C, Bioanalytical Systems Inc.) was employed. Applied potential was 0.75 volt vs Ag/AgCl as reference. Mobile phase (one liter) contained 2.1 g heptanesulfonic acid, 3.5 ml triethylamine, 3 ml phosphoric acid, 0.1 g NaEDTA and 170 ml acetonitrile. The retention time for dopamine was about 7.5 min [37].

### Western blot analysis of relevant proteins

After decapitation, dissected SN was homogenized with a sonicator in 40 µl ice-cold protease inhibitor cocktail (Calbiochem, San Diego, CA). The lysates were centrifuged at 12,000× g at 4°C for 30 min, the supernatant was stored at −80°C. Protein samples were run on sodium dodecyl sulfate (SDS)-polyacrylamide gel electrophoresis (8–13.5%) and then transferred onto a nitrocellulose membrane (Bio-Rad, Hercules, CA) at 80 V for 2 hours (h). Blots were probed with a monoclonal antibody against heme oxygenase −1 (HO-1, 32Kd; 1∶1000; StressGen, Victoria, CA), Atg7 (1∶1000, Thermo Scientific, Rockford, IL), LC3 (1∶1000; Novus, Littleton, CO), caspase 9 (1∶1000; Cell Signaling Tech., Danvers, MA), tyrosine hydroxylase (TH; 1∶3000; Chemicon, Temecula, CA), cytochrome c oxidase (1∶1000; Molecular Probes, Eugene, OR), caspase 12 (1∶1000; Exalpha Biologicals, Shirley, MA) and α-synuclein (1∶1000; BD Transduction Lab., Lexington, KY) at room temperature for 2 h. After primary antibody incubation, the membrane was washed and incubated with horseradish peroxidase-conjugated secondary IgG (1∶3000; Chemicon, Temecula, CA) for 1 h at room temperature. The immunoreaction was visualized by Amersham enhanced chemiluminescence (Amersham Pharmacia Biotech, Piscataway, NJ). After this detection, the bound primary and secondary antibodies were stripped by incubating the membrane in stripping buffer (100 mM 2-mercaptoethanol, 2% SDS) at 50°C for 45 min. The membrane was reprobed with a mouse β-actin antibody (1∶5000; Millipore, Bedford, MA). The densities of blots were analyzed using a scanning densitometer. Results were obtained by calculating the density using ImageJ software (NIH, Bethesda, MD) and reported as relative optical density of the specific proteins. All the bands on blots were corrected by corresponding β-actins shown as relative protein density (arbitrary units) for further statistic analysis.

### Immunofluorescence staining

Rats were transcardially perfused with 0.9% saline followed by fixative consisting of 4% paraformaldehyde in 0.1 M phosphate-buffered saline (PBS). Brains were removed and placed in 30% sucrose-PBS overnight and frozen-sectioned coronally at 30 µm thickness. After fixation, permeabilization and blocking, sections were incubated overnight at 4°C with primary antibodies including α-synuclein (1∶100), LC3 (1∶100) and succinate dehydrogenase (1∶100), followed by incubation with secondary antibodies, including rhodamine (1∶500) and fluorescein isothiocyanate (1∶500) for 1 h at room temperature. Nuclei were labeled with 4′, 6-diamidino-2-phenylindole (1 mg/ml) for 10 min at room temperature. After final washes and mounting, fluorescence images of the fixed cultures were viewed with a fluorescence laser-scanning confocal microscope (Olympus FV10i, Center Valley, PA).

### Statistics

The results of Western blot assays of time-dependent effects of MPP^+^ were analyzed by one-way analysis of variance (one-way ANOVA) and followed by the LSD test as post-hoc method. The results of Western blot assays of MPP^+^ plus Atg7 siRNA transfection were analyzed by Kruskal-Wallis test and followed by Mann-Whitney U test as post-hoc analysis with Bonferroni correction. The results of lipid peroxidation and striatal dopamine content were analyzed by *t*-test. P value less than 0.05 was considered as statistically significant.

## Results

### MPP^+^-induced oxidative injury in nigrostriatal dopaminergic system of rat brain

**Figure 1:**
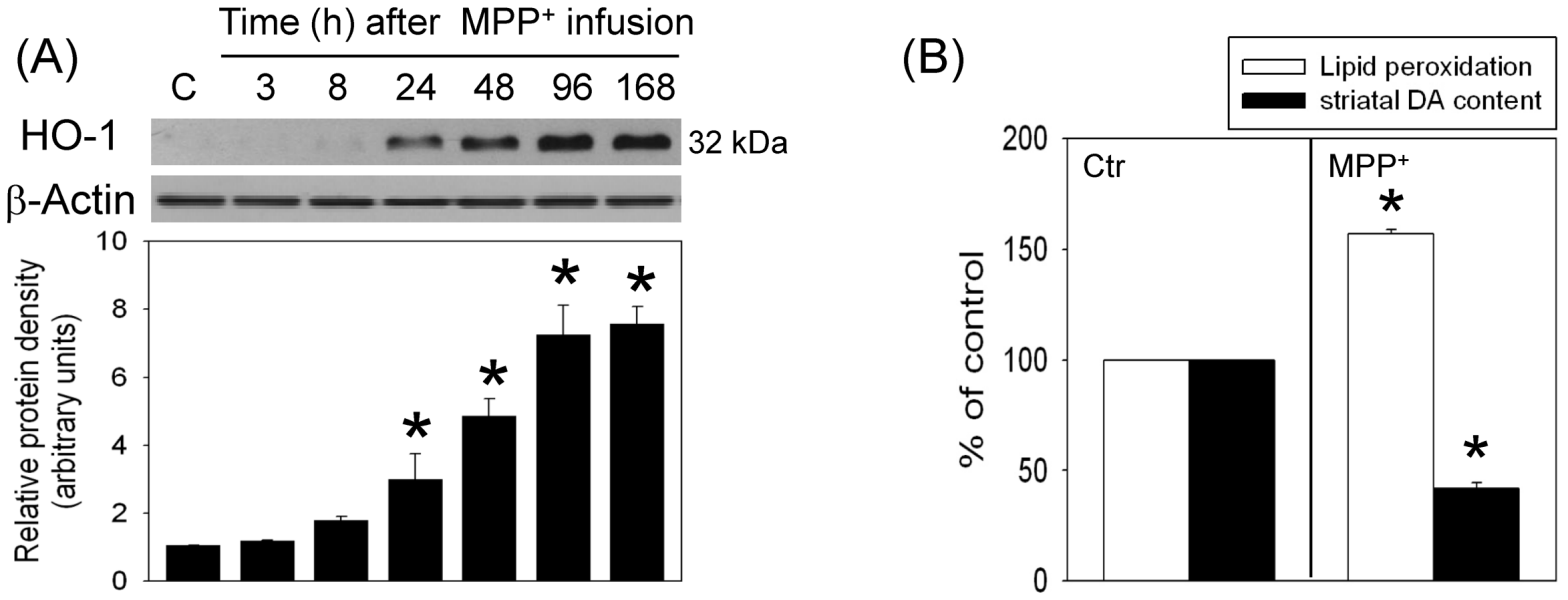
MPP^+^ induced oxidative injury in the nigrostriatal dopaminergic system of rat brain. Intranigral infusion of MPP^+^ (3 µg/µl) was performed in the anesthetized rats. Rats were sacrificed at the indicated times. (A) Time-dependent effects of MPP^+^ on heme oxygenase (HO)-1 level in the substantia nigra (SN) were studied using Western blot assay. Each lane contained 25 µg for all experiments. Graphs show statistical results from relative optical density of bands on the blots estimated by Image J software. Values are the mean±S.E.M. (n = 3/group). *P<0.05 in the MPP^+^-treated groups compared with the control groups by one-way analysis of variance (one-way ANOVA) and followed by the LSD test as post-hoc method. (B) Seven days after MPP^+^ infusion, lipid peroxidation in SN was reported as relative fluorescence unit (RFU) and striatal dopamine content was detected by HPLC-ECD. Values are the mean ± S.E.M. (n = 4/group). *P<0.05 in the MPP^+^-treated groups compared with the control groups by *t*-test.

To study the oxidative mechanism underlying MPP^+^-induced neurotoxicity, MPP^+^ was locally infused in the SN of anesthetized rats; HO-1 level, a sensor of oxidative stress was evaluated using Western blot assay. Our data showed that intranigral infusion of MPP^+^ increased HO-1 levels in a time-dependent manner; significant HO-1 elevation was observed 24 h to 7 d after MPP^+^ infusion (Figure 1A). Compared with intact SN, lipid peroxidation, a hallmark that free radicals attack cells, was elevated in the MPP^+^-infused SN (n = 4) 7 d after the infusion. At the same time, we observed reduction in striatal dopamine level ipsilateral to the MPP^+^-infused SN (Figure 1B), indicating that MPP^+^ deteriorated the nigrostriatal dopaminergic transmission in rat brain.

### MPP^+^-induced autophagy

**Figure 2:**
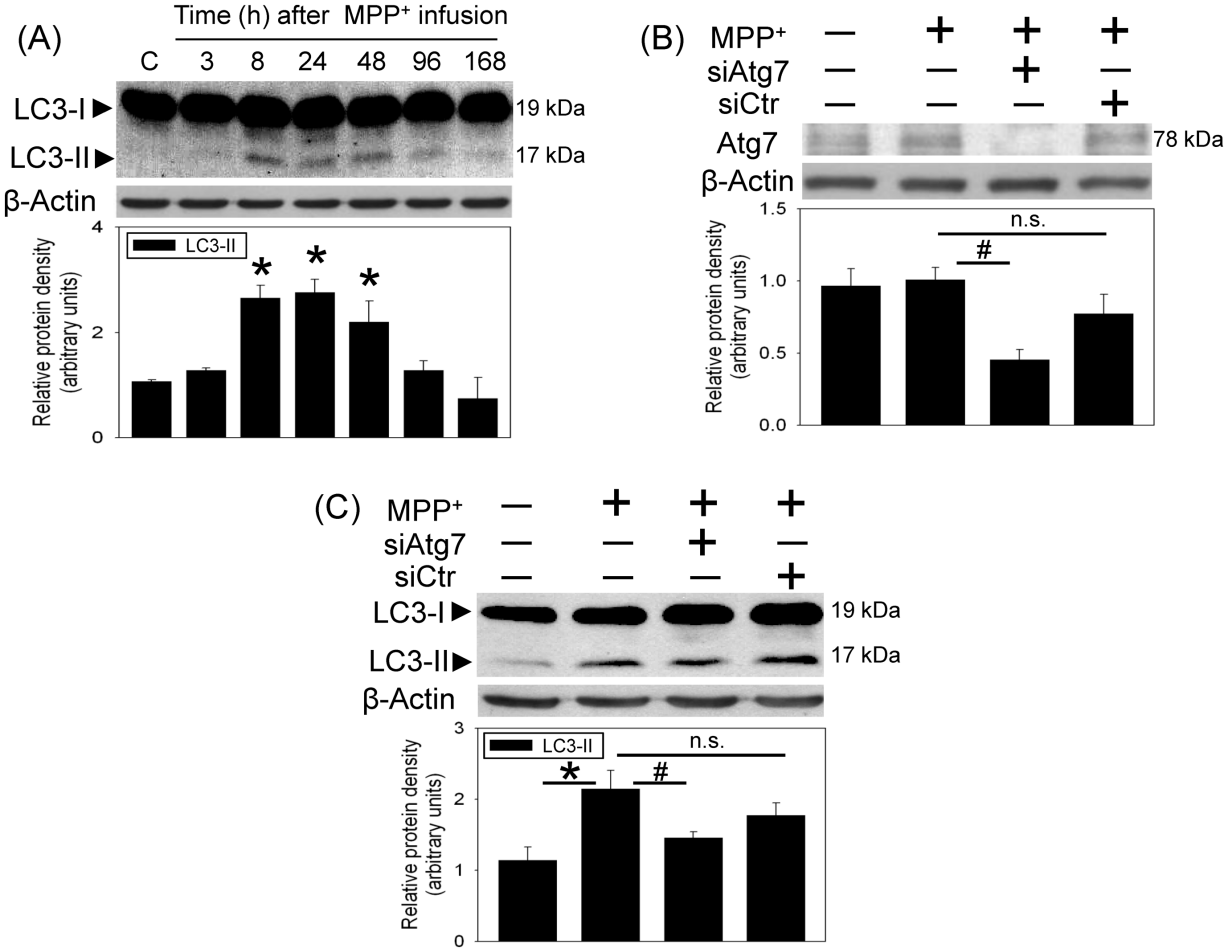
MPP^+^ induced autophagy in the nigrostriatal dopaminergic system of rat brain. Intranigral infusion of MPP^+^ (3 µg/µl) was performed in the anesthetized rats. Rats were sacrificed at the indicated times. (A) Time-dependent effects of MPP^+^ on LC3-II level in the substantia nigra (SN) were studied using Western blot assay. Graphs show statistical results from relative optical density of bands on the blots estimated by Image J software. Values are the mean±S.E.M. (n = 3/group). *P<0.05 in the MPP^+^-treated groups compared with the control groups by one-way analysis of variance (one-way ANOVA) and followed by the LSD test as post-hoc method. (B and C) Intranigral infusion of Atg7siRNA was performed 4 days prior to intranigral infusion of MPP^+^ (3 µg/µl) in chloral hydrate-anesthetized rats. Rats were sacrificed 30 h after intranigral infusion of MPP^+^. The levels of Atg7protein (B) and LC3-II (C) levels in SN were measured using Western blot assay. Each lane contained 25 µg protein for the experiments. Graphs show statistical results from relative optical density of bands on the blots estimated by Image J software. Values are the mean±S.E.M. (n = 4/group). *P<0.05 in the MPP^+^-infused SN compared with the intact SN, # P<0.05 in Atg7siRNA transfected and MPP^+^-infused SN compared with MPP^+^-infused SN, n.s. for not significant by Kruskal-Wallis test and followed by Mann-Whitney U test as post-hoc method.

**Figure 3:**
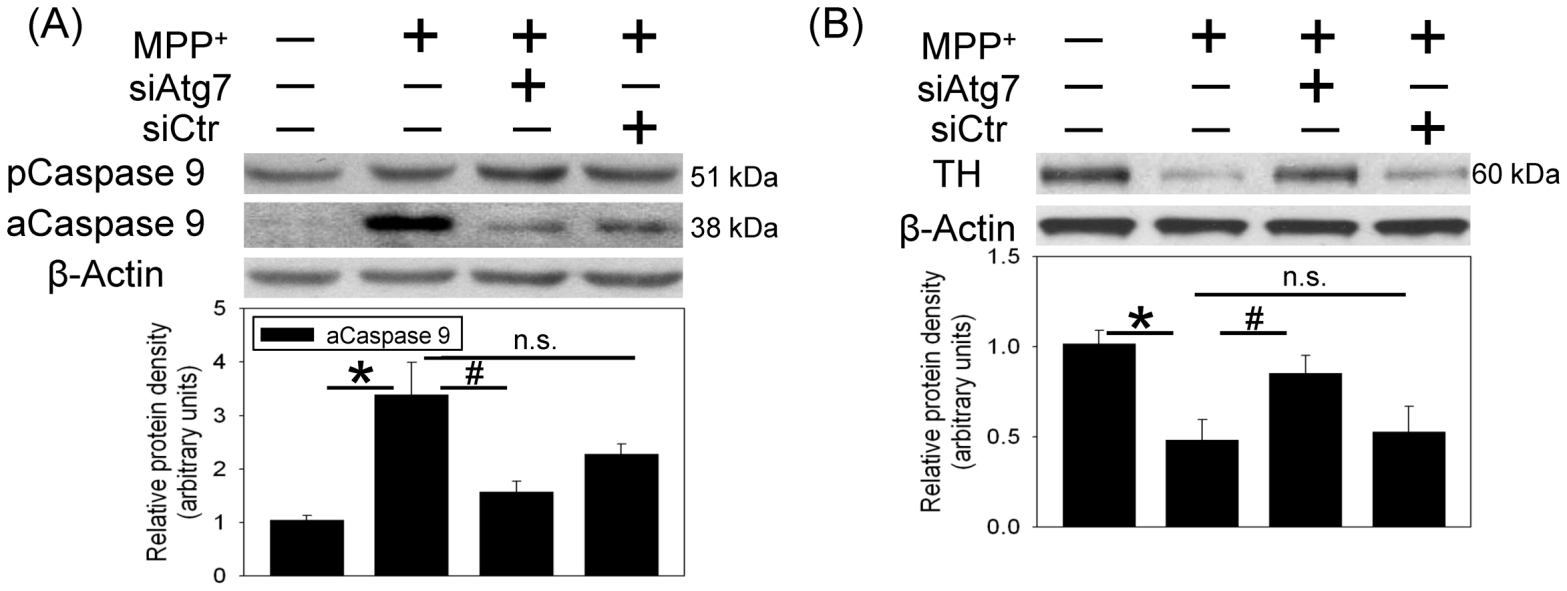
*In vivo* Atg7siRNA transfection attenuated MPP^+^-induced caspase 9 activation and tyrosine hydroxylase (TH) reduction in the nigrostriatal dopaminergic system of rat brain. Intranigral infusion of Atg7siRNA was performed 4^+^ (3 µg/µl) in the anesthetized rats. Rats were sacrificed 30 h after intranigral infusion of MPP^+^. (A) Caspase 9 in the substantia nigra (SN) was measured using Western blot assay. Each lane contained 25 µg protein for the experiments. Values are the mean±S.E.M. (n = 4/group). *P<0.05 in the MPP^+^-infused SN compared with the intact SN, # P<0.05 in Atg7siRNA transfected and MPP^+^-infused SN compared with MPP^+^-infused SN, n.s. for not significant by Kruskal-Wallis test and followed by Mann-Whitney U test as post-hoc method. (B) TH in the substantia nigra (SN) was measured using Western blot assay. Each lane contained 3 µg protein for the experiments. Graphs show statistical results from relative optical density of bands on the blots estimated by Image J software. Values are the mean±S.E.M. (n = 3/group). *P<0.05 in the MPP^+^-treated groups compared with the control groups by one-way analysis of variance (one-way ANOVA) and followed by the LSD test as post-hoc method.

The involvement of autophagy in MPP^+^-induced neurotoxicity was investigated by measuring the gel shift of LC3-II (17 kDa) from LC3-I (19 kDa). Western blot assay showed that MPP^+^ increased LC3-II levels in the infused SN 8 h after intranigral infusion of MPP^+^. The MPP^+^-induced LC3-II elevation was maintained for 48 h and gradually decreased at 96 h after MPP^+^ infusion (Figure 2A). To verify the role of autophagy in MPP^+^-induced autophagy, *in vivo* Atg7siRNA transfection was performed by local infusion of Atg7siRNA in the SN for 4 d prior to MPP^+^ infusion. In contrast to the MPP^+^-induced elevation in LC3-II, intranigral infusion of MPP^+^ did not alter Atg7 level. However, Atg7siRNA transfection profoundly attenuated the Atg7 levels in MPP^+^-infused SN (Figure 2B). At the same time, Atg7siRNA transfection decreased MPP^+^-induced elevation in LC3-II levels (Figure 2C), indicating that Atg7 silencing prevented MPP^+^-induced autophagy. Furthermore, MPP^+^-induced caspase 9 activation (Figure 3A) and reduction in TH (Figure 3B) were diminished in the Atg7siRNA-transfected SN, indicating that autophagy is pro-death in the MPP^+^-induced oxidative injury.

**Figure 4:**
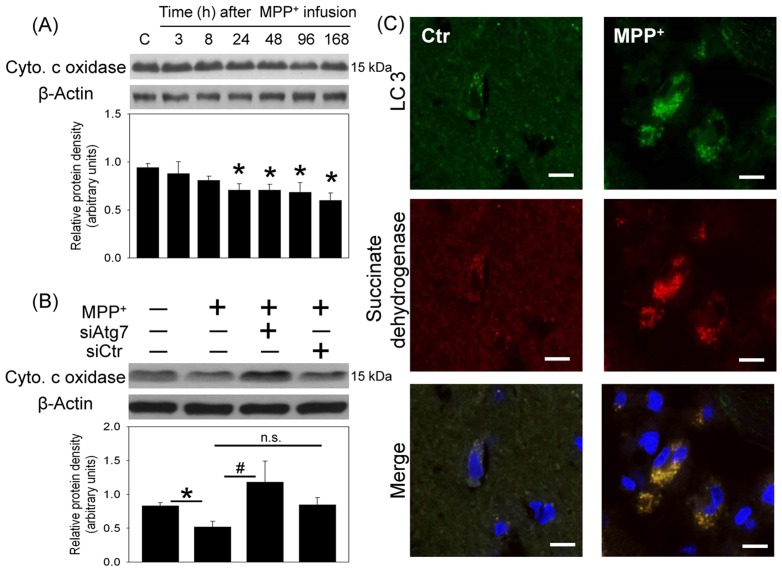
Effect of MPP^+^ on mitochondria content in the nigrostriatal dopaminergic system of rat brain. Intranigral infusion of MPP^+^ (3 µg/µl) was performed in the anesthetized rats. Rats were sacrificed at the indicated times. (A) Time-dependent effects of MPP^+^ on cytochrome c oxidase levels in the substantia nigra (SN) were studied using Western blot assay. Values are the mean±S.E.M. (*n* = 5/group). *P<0.05 in the MPP^+^-treated groups compared with the control groups by one-way analysis of variance (one-way ANOVA) and followed by the LSD test as post-hoc method. (B) Intranigral infusion of Atg7siRNA was performed 4 days prior to intranigral infusion of MPP^+^ (3 µg/µl) in chloral hydrate-anesthetized rats. Rats were sacrificed 30 h after intranigral infusion of MPP^+^. The levels of cytochrome c oxidase in SN were measured using Western blot assay. Each lane contained 25 µg protein for the experiments. Graphs show statistical results from relative optical density of bands on the blots estimated by Image J software. Values are the mean±S.E.M. (n = 4/group). *P<0.05 in the MPP^+^-infused SN compared with the intact SN, # P<0.05 in Atg7siRNA transfected and MPP^+^-infused SN compared with MPP^+^-infused SN, n.s. for not significant by Kruskal-Wallis test and followed by Mann-Whitney U test as post-hoc method. (C) Representative immunostaining shows co-localization of LC3 (green) and succinate dehydrogenase (red) in the MPP^+^-infused SN. Nuclear staining was indicated by blue. Results were repeated with independent experiments.

Two proposed targets of autophagy, including mitochondria and α-synuclein were investigated. First, MPP^+^-induced mitochondria loss was evident by the observation that cytochrome c oxidase levels (a mitochondria specific protein) were time-dependently reduced in the infused SN (Figure 4A). *In vivo* Atg7siRNA transfection was found to reverse MPP^+^-induced reduction in cytochrome c oxidase in the transfected SN (Figure 4B). Furthermore, our immunostaining study demonstrated that LC3 immunofluorescence was co-localized with that of succinate dehydrogenase (a biomarker of mitochondria) 24 h after MPP^+^ infusion, indicating that autophagy may engulf mitochondria and thus results in mitochondria loss (Figure 4C).

**Figure 5:**
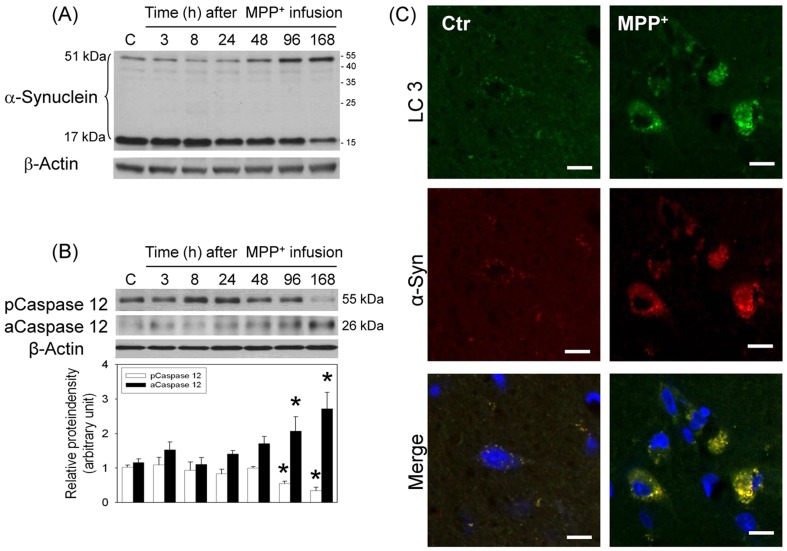
Effect of MPP^+^ on α-synuclein aggregation in the nigrostriatal dopaminergic system of rat brain. Intranigral infusion of MPP^+^ (3 µg/µl) was performed in the anesthetized rats. Rats were sacrificed at the indicated times. (A) Representative data show time-dependent effects of MPP^+^ on α-synuclein aggregation in the substantia nigra (SN) using Western blot assay. Results were repeated with independent experiments. (*n* = 3) (B) Time-dependent effects of MPP^+^ on caspase 12 activation in SN were studied using Western blot assay. Each lane contained 25 µg protein for the experiments. Graphs show statistical results from relative optical density of bands on the blots estimated by Image J software. Values are the mean±S.E.M. (n = 5/group). *P<0.05 in the MPP^+^-treated groups compared with the control groups by one-way analysis of variance (one-way ANOVA) and followed by the LSD test as post-hoc method. (C) Representative immunostaining shows co-localization of LC3 (green) and α-synuclein (red) in the MPP^+^-infused SN. Nuclear staining was indicated by blue. Results were repeated with independent experiments.

**Figure 6:**
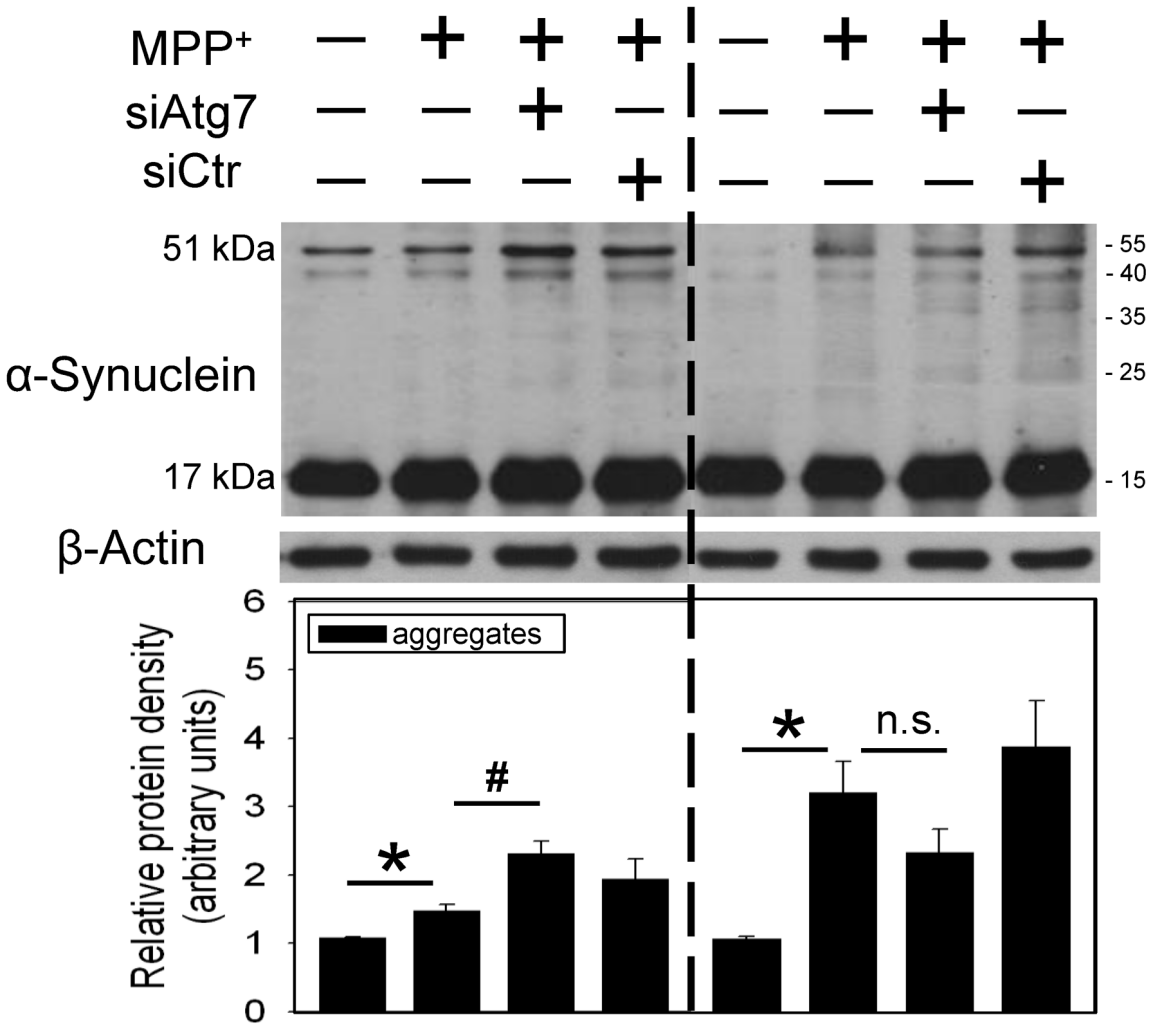
Differential effects of *in vivo* Atg7siRNA transfection on MPP^+^-induced α-synuclein aggregation in the nigrostriatal dopaminergic system of rat brain. Intranigral infusion of Atg7siRNA was performed 4 days prior to intranigral infusion of MPP^+^ (3 µg/µl) in the anesthetized rats. Rats were sacrificed 30 h after intranigral infusion of MPP^+^. The levels of α-synuclein aggregation in the substantia nigra (SN) were measured using Western blot assay. Each lane contained 25 µg protein for the experiments. Graphs show statistical results of α-synuclein (51 kd) from relative optical density of bands on the blots estimated by Image J software. Values are the mean±S.E.M. (n = 4). *P<0.05 in the MPP^+^-infused SN compared with the intact SN, # P<0.05 in Atg7siRNA transfected and MPP^+^-infused SN compared with MPP^+^-infused SN, n.s. for not significant by Kruskal-Wallis test and followed by Mann-Whitney U test as post-hoc method.

In addition, intranigral infusion of MPP^+^ time-dependently decreased α-synuclein monomer levels (17 kDa) and increased α-synuclein aggregation (51 kDa, Figure 5A). At the same time, a time-dependent reduction in procaspase 12 and elevation in active caspase 12 were observed 4–7 d after intranigral infusion of MPP^+^ (Figure 5B), indicating that MPP^+^-induced α-synuclein aggregation may induce ER stress. Using immunostaining techniques, co-localization of LC3 immunofluorescence and α-synuclein immunofluorescence was demonstrated 24 h after intranigral infusion of MPP^+^ (Figure 5C), suggesting that autophagy engulfs α-synuclein as well. However, differential effects of *in vivo* Atg7 siRNA transfection on α-synuclein aggregation were observed. When MPP^+^-induced α-synuclein aggregation was mild (left part of Figure 6), *in vivo* Atg7siRNA transfection was effective for enhancing MPP^+^-induced α-synuclein aggregation, indicating that autophagy significantly degraded α-synuclein aggregates. In contrast, in the presence of profound MPP^+^-induced α-synuclein aggregation, Atg7siRNA transfection insignificantly modulated the MPP^+^-induced α-synuclein aggregation, suggesting that autophagy was unable to efficiently remove the α-synuclein aggregates when the levels of α-synuclein aggregates were relatively high (right part of Figure 6).

## Discussion

Both our previous [38] and the present studies showed that intranigral infusion of MPP^+^ induced oxidative injury in the nigrostriatal dopaminergic system of rat brain. In addition to apoptosis, the present study delineated the prodeath mechanism of autophagy in CNS neurodegeneration. Our *in vivo* study was the first to demonstrate the dynamic changes in MPP^+^-induced LC3-II levels by intranigral infusion of MPP^+^ in rat brain. We found that MPP^+^ significantly increased LC3-II levels followed by a gradual reduction in LC3-II level 4–7 d after MPP^+^ infusion. Previously, we reported a similar delayed reduction in LC3-II levels in the hypoxia-treated SH-SY5Y cells [9] and the kainate-treated hippocampus of mouse brain [12]. Furthermore, our previous *in vitro* study and others have demonstrated that bafilomycin A1 (an inhibitor of autolysosome formation) was capable of potentiating LC3-II levels [8]–[9], [22]. Therefore, the mechanism for the delayed reduction in LC3-II levels may be due to the degradative flux of autophagy because formation of autolysosomes has been suggested to degrade the LC3-II in the inner membrane of autophagosomes [39]–[40]. Indeed, a corresponding elevation in cathepsin B (a lysosomal enzyme) was observed in the MPP^+^-infused SN (our unpublished data). These data indicate a consistently active autophagic activity in the MPP^+^-infused SN.

Defective mitochondria, oxidative stress and energy depletion are part of the vicious cycle in the etiology of Parkinsonism [13], [17]. MPP^+^, by targeting mitochondrial complex I, is known to damage mitochondria and result in oxidative stress [9], [13], [19]–[20]. Our *in vivo* study confirmed the involvement of oxidative stress in MPP^+^-induced neurotoxicity in 2 aspects: MPP^+^-induced lipid peroxidation and HO-1 elevation, a sensor of oxidative stress [41]. In addition to damaging mitochondria, several studies have reported MPP^+^-induced mitochondrial loss [19]–[20]. Our study supports this notion by MPP^+^-induced decreases in cytochrome c oxidase in the infused SN. The fate of damaged mitochondria has been proposed by autophagy-induced degradation [23]–[24]. Indeed, we demonstrated co-localization of LC3 and succinate dehydrogenase (a mitochondria biomarker) as well as Atg7siRNA transfection-induced attenuation of MPP^+^-induced cytochrome c oxidase reduction. These data indicate a neuroprotective role of autophagy. However, a pro-death role of autophagy was simultaneously observed by Atg7siRNA-induced mitigation of MPP^+^-induced TH reduction and apoptosis. An explanation of the conflicted data is proposed: when the cell is challenged by a mild stress or during the beginning of insults, mitochondrial biogenesis is reportedly induced [42]–[43]; this effect may compensate for the loss of damaged mitochondria. Accordingly, autophagy appears to be neuroprotective by eliminating damaged mitochondria [9], [13], [19]–[20]. Nevertheless, under lethal conditions, such as a prolonged MPP^+^-induced oxidative stress, both overdriven autophagy and impaired mitochondria biogenesis [20] may result in insufficient amount of intact mitochondria which lead to cell death in the MPP^+^-affected SN.

Several forms of α-synuclein have been identified; oligomers and protofibrillar forms are reportedly more neurotoxic than the monomeric forms [44]. Our previous studies have shown α-synuclein aggregation by transition metals in SN [45]–[46] and neurotoxins in hippocampus [12]. The present study consistently showed time-dependent elevation in α-synuclein aggregates with a reduction in α-synuclein monomers in the MPP^+^-infused SN. Different autophagy-lysosome systems have been proposed for the degradation of α-synuclein. α-Synuclein monomers are reportedly degraded by autophagy and chaperone-mediated autophagy [33], [47]–[48]. As to the degradation of α-synuclein aggregates, some studies suggest autophagy [29], [34] but the others do not support this notion [49]. The inconsistency of degradation may be due to the levels of α-synuclein aggregates. From our immunofluorescence study demonstrating co-localization of α-synuclein with LC3, α-synuclein is suggested to be degraded by autolysosomes [33]–[34], [48]. However, Atg7siRNA transfection differentially modulated α-synuclein aggregation. In the presence of mild α-synuclein aggregation in MPP^+^-infused SN, the autophagic clearance of α-synuclein aggregates was evident by Atg7siRNA-induced enhancement of α-synuclein aggregates. In contrast, autophagy continued but was unable to significantly degrade α-synuclein aggregates if there was a significant amount of α-synuclein aggregates. These findings may support the clinical significance of autophagy in that at the initial disease stage with low synuclein aggregation, autophagy is induced to remove protein aggregates [29], [33], [48]; but, when the disease progresses and more aggregates are produced, autophagy was unable to efficiently remove the overwhelming amount of α-synuclein aggregates [28]. Accordingly, α-synuclein aggregates may accumulate and thus induce unfolded protein response pathways, i.e.ER stress [50]; indeed, our data showed a delayed activation of caspase 12, an endoplasmic reticulum (ER)-specific enzyme [51] in the MPP^+^-affected SN. Other neurotoxic mechanisms of α-synuclein aggregates include inhibiting autophagy [52] and ubiquitin-proteasome system [53]; this vicious cycle appears to aggravate the α-synuclein aggregation during the progress of CNS neurodegenerative diseases.

In conclusion, autophagy appears to play a critical role in MPP^+^-induced neurodegeneration of the nigrostriatal dopaminergic system of rat brain. Our study further suggests that autophagy engulfs mitochondria and results in mitochondrial loss but the benefits of autophagy depend on the level of residual intact mitochondria. Similarly, autophagy sequesters α-synuclein aggregates but the benefits of autophagy depend on the levels of α-synuclein aggregates in the nigrostriatal dopaminergic system of rat brain.
